# Characterisation of the Ovine *KRTAP36-1* Gene in Chinese Tan Lambs and Its Impact on Selected Wool Traits

**DOI:** 10.3390/ani15152265

**Published:** 2025-08-01

**Authors:** Lingrong Bai, Huitong Zhou, Jinzhong Tao, Guo Yang, Jon G. H. Hickford

**Affiliations:** 1 International Wool Research Institute, Faculty of Animal Science and Technology, Gansu Agricultural University, Lanzhou 730070, China; lingrong.bai@lincolnuni.ac.nz (L.B.); huitong.zhou@lincoln.ac.nz (H.Z.); tao_jz@nxu.edu.cn (J.T.); 2 Gene-Marker Laboratory, Faculty of Agriculture and Life Sciences, Lincoln University, Lincoln 7647, New Zealand; 3 Yellow River Estuary Tan Sheep Institute of Industrial Technology, Dongying 257400, China; yangguo@lzb.ac.cn; 4 College of Animal Science and Technology, Ningxia University, Yinchuan 750021, China; 5 State Key Laboratory of Ecological Safety and Sustainable Development in Arid Lands, Northwest Institute of Eco-Environment and Resources, Chinese Academy of Sciences, Lanzhou 730000, China

**Keywords:** keratin-associated protein, KAP36-1, fibre curvature, fine wool, heterotypic hair fibres, Chinese Tan lambs

## Abstract

Wool has distinctive biological, physical, and chemical properties that contribute to its value both for the sheep and in global fibre and textile markets. Its fibres are primarily composed of proteins, principally keratin and keratin-associated proteins (KAPs). To better comprehend the genes that underpin key wool traits, this study examined the keratin-associated protein 36-1 gene (*KRTAP36-1*) in Chinese Tan lambs. We identified three previously reported alleles of the gene (named *A*, *B* and *C*) that were present in the lambs studied, with genotype frequencies as follows: 2.0% (*n* = 5; *AA*), 6.9% (*n* = 17; *AB*), 13.8% (*n* = 34; *AC*), 8.9% (*n* = 22; *BB*), 33.4% (*n* = 82; *BC*) and 35.0% (*n* = 86; *CC*). The frequencies of the individual alleles in the Chinese Tan lambs were 12.4%, 29.1% and 58.5% for alleles *A*, *B* and *C*, respectively. The three alleles were in Hardy–Weinberg Equilibrium. In an association analysis, it was revealed that allele *C* was associated with variation in the mean fibre curvature of the fine wool of the Chinese Tan lambs, but this association was not observed in their heterotypic hair fibres. This finding suggests that *KRTAP36-1* might be differentially expressed in the wool follicles that produce the two fibre types, and that along with other *KRTAP* genes, it may be involved in determining fibre curvature and the distinctive curly coat of the lambs.

## Simple Summary

Found predominantly in the Ningxia Hui Autonomous Region, Tan sheep are a dual-purpose breed unique to China. Their population is believed to number in the millions, and they are renowned and valued by the Chinese for their meat and distinctive curly fleece at approximately one month of age. There are limits to our knowledge of the genetic mechanisms that underpin their fibre development and that contribute to their fleece characteristics. To better understand the formation of curly fibres, this study focused on the wool keratin-associated protein gene *KRTAP36-1* in Tan lambs. Sequence variation in this gene was found to be associated with mean fibre curvature in the fine wool fibres of the breed, but not in the heterotypic hair fibres which have a larger fibre diameter.

## 1. Introduction

Wool is an important animal fibre and an income source for sheep farmers, along with pelts, milk and meat [1]. Textiles made of wool yarns are found globally in clothing markets and valued for their unique natural properties [2]. These properties include excellent breathability, superior moisture management capabilities, effective thermoregulation, inherent fire resistance, notable elasticity and biodegradability. Collectively, these attributes make wool an increasingly preferred sustainable fibre choice across a broader range of textile applications [2]. However, the wool fibres used in these textiles can be variable. This affects the quality of items that can be created with the wool, as well as the quality and characteristics of pelts used in the fur trade. The mechanisms that underly wool fibre variability are not well understood, which limits both the uses of wool and its commercial value in fibre and yarn markets [3].

Wool is a proteinaceous fibre, composed primarily of two types of protein: helical keratins and the more amorphous keratin-associated proteins (KAPs) that form a matrix bonded to the keratins [4]. If wool consistency is to be improved to enable market development, it is important to better understand the genes that encode the wool proteins and how they might affect fibre and fleece traits. The KAPs are described as a broad grouping of proteins [4,5]. Based on their amino acid composition, they can be split into three groups. These are termed the high-sulphur (HS) proteins, the ultra-high sulphur (UHS) proteins, and the high-glycine-and-tyrosine (HGT) proteins [6]. Numerous KAP genes, designated *KRTAP*s, have been identified in animals [7,8,9], although the total number across species has not yet been fully established. Humans have a well-defined set of eighty-nine *KRTAPs* [10,11,12]. These human *KRTAPs* are classified into twelve HS-KAP families (containing 25 *KRTAPs*), six UHS-KAP families (containing 47 *KRTAP*s) and seven HGT-KAP families (containing 17 *KRTAP*s) [10,11,12]. A recent study has reported a total of 102 *KRTAPs* in sheep and 98 in goats, highlighting the diversity of these genes in small ruminants [13].

Of the known ovine genes, *KRTAP36-1* is a member of the HGT-KAP group. This gene has been described previously in New Zealand Southdown × Merino-cross lambs, with four single nucleotide polymorphisms (SNPs) and three unique nucleotide sequences (named *KRTAP36-1 A*, *B* and *C*) having been identified. Allele *B* was associated with an increase in the prickle factor (PF; the percentage of fibres within a sample that have a diameter greater than 30 microns) of the Southdown × Merino-cross wool [14].

Tan sheep are an indigenous Chinese breed, predominantly raised in the Ningxia Hui Autonomous Region of northwestern China [15]. They are known in both domestic and foreign markets for their highly curved lamb ‘fur’ at approximately one month old [16]. Higher curvature fibre traps a larger volume of air between the fibres, giving it increased insulative value as a pelt.

In this study, the variability of *KRTAP36-1* was investigated in Chinese Tan lambs to determine whether there was variation in the gene comparable to that reported previously, and if found, whether it was related to wool fibre traits in the breed.

## 2. Materials and Methods

### 2.1. The Chinese Tan Sheep Investigated and Wool Trait Measurement

The study comprised 246 single-born Chinese Tan lambs (127 males and 119 females) from ten recorded sire-lines. Wool samples were gathered from the mid-side region of the lambs at ‘Er-mao’ (day thirty-five post-partum). For these samples, the fine wool fibres and heterotypic hair fibres were physically separated based on the visible difference in their fibre diameter and length. This separation method involved pressing the base of all fibres against a flannel board while using the other hand to isolate the longer heterotypic hair fibres. This process is repeated numerous times to ensure all the heterotypic hair fibres of the wool sample are isolated from the fine wool fibres. A minimum of 1000 fibres were collected from each sample for analysis.

Measurements of the mean fibre diameter (MFD), fibre diameter standard deviation (FDSD), coefficient of variation in the fibre diameter (CVFD) and mean fibre curvature (MFC) were obtained for fine wool fibres and heterotypic hair fibres. Pastoral Measurements Limited (Timaru, New Zealand) evaluated the fine wool samples, while the hair samples were measured by the New Zealand Wool Testing Authority (NZWTA, Napier, New Zealand) using the International Wool Textile Organisation (IWTO)—standardised Laserscan method IWTO-12-2012. The CVFD was calculated from MFD and FDSD by dividing FDSD by MFD and multiplying by one hundred to express it as a percentage.

Venous blood samples from each of the Tan lambs were collected onto filter paper (TFN paper; Munktell Filter AB, Falun, Sweden). These papers were air-dried and stored in the dark at room temperature until subsequently required. Genomic DNA from the blood sample was prepared for PCR amplification as per Gong et al. [14].

### 2.2. PCR Amplification and Single-Strand Conformation Analysis

Polymerase chain reaction amplification of the *KRTAP36-1* target sequence was conducted using the same primers as Gong et al. [14]. The amplifications were undertaken in a 15-μL reaction volume that contained the purified genomic DNA on the TFN paper. The reaction mixture was 150 μM for each of the four dNTPs (Bioline, London, UK) and 0.25 μM for each primer. It contained 2.5 mM Mg^2+^, 0.5 U of Taq DNA polymerase (Qiagen, Hilden, Germany), and 1× the reaction buffer supplied with the enzyme. The thermal profile for amplification included an initial denaturation for 2 min at 94 °C. This was then followed by 35 cycles of 30 s at 94 °C, 30 s at 60 °C and 30 s at 72 °C, and with a final extension step for 5 min at 72 °C. Thermal cycling was conducted in S1000 thermal cyclers (Bio-Rad; Hercules, CA, USA).

The PCR amplicons produced for each lamb were analysed using a single-strand conformation polymorphism (SSCP) approach with the amplicon at limiting dilution. In this analysis, a 0.7-μL aliquot of each amplicon from the PCR reactions is mixed with 7 μL of gel loading dye (0.025% bromophenol blue, 0.025% xylene-cyanol, 98% formamide, 10 mM EDTA). After denaturation at 95 °C for 5 min, the samples are rapidly cooled on wet ice to prevent re-annealing and maintain the DNA in a single-stranded state, allowing the formation of secondary structures. The denatured samples were then immediately loaded on 14% acrylamide: bisacrylamide (37.5:1; Bio-Rad) gels (16 cm × 18 cm) and electrophoresed in 0.5× TBE buffer at 300 V and 11 °C for 19 h using Protean II xi cells (Bio-Rad). Upon completion of the electrophoresis, the gels were removed from the apparatus, and fixed and stained for 10 min in a solution containing 10% ethanol, 0.5% acetic acid, and 0.2% silver nitrate. They were then rinsed with distilled water and placed in a developing solution (3% NaOH and 0.1% HCOH) until dark-staining single-strand DNA bands from the formation of stable secondary structures appeared against the yellow background. When the bands became clearly visible, development was stopped by removing the developing solution and by the addition of a solution containing 10% ethanol and 0.5% acetic acid. This method has been described by Byun et al. [17]. Selected sheep DNA samples representing the different *KRTAP36-1* alleles identified previously by Gong et al. [14] were obtained from that author and amplified. These PCR amplicons were denatured and run on the gels as reference sequences for allele identification.

### 2.3. Statistical Analyses

Hardy–Weinberg Equilibrium (HWE) was tested using an online calculator (www.had2know.org/academics/hardy-weinberg-equilibrium-calculator-3-alleles.html, accessed on 5 June 2025). Statistical analyses were performed using Minitab version 16 (Minitab Inc., State College, PA, USA). The effect of nucleotide sequence variation in *KRTAP36-1* on the four wool traits measured was evaluated using general linear models (GLMs) to evaluate the effect of the presence or absence (coded as “1” or “0”) of the three alleles of the gene (*A*, *B* and *C)*, on the four fibre traits. The models incorporated both sire and lamb gender, because sire was identified to have an influence on all the traits, while gender was identified as a factor that impacted some of the traits. The model used was Y_ijk_ = μ + V_i_ + Gender_j_ + Sire_k_ + e_ijk_, where Y_ijk_ is the phenotype (for either MFD, CVFD, FDSD, or MFC) of the ijk^th^ lamb, μ = is the arithmetic mean for the characteristic, V_i_ is the effect of the i^th^ allele (presence or absence), Gender_j_ is the fixed effect of the j^th^ gender, Sire_k_ is the random effect of the k^th^ sire, and e_ijk_ is the random residual effect. When *p* < 0.05, the associations were considered to be significant.

## 3. Results

### 3.1. Genetic Variation in KRTAP36-1 in Chinese Tan Lambs

The PCR-SSCP typing system revealed banding patterns representing three DNA alleles of *KRTAP36-1* in Chinese Tan lambs. Upon analysis and comparison of the PCR-SSCP gels, these were confirmed to correspond to the alleles (*A*, *B* and *C*) previously described by Gong et al. [14] in the Southdown × Merino-cross. Six genotypes of these allele sequences were observed in the 246 Chinese Tan lambs (Table 1). The distribution of the three alleles was assessed for HWE, and the result indicated no significant deviation from equilibrium (*p* = 0.886).

### 3.2. Associations of KRTAP36-1 Variation and Wool Traits

Mean fibre curvature was associated with *KRTAP36-1* variation for the fine wool fibres (Table 2), but no associations were detected for the heterotypic hair fibres. Chinese Tan Lambs with the allele *C* had fine wool fibres with greater MFC than those without the allele. The CVFD of fine wool fibres from sheep with allele *B* tended to be greater (*p* < 0.1), while for the heterotypic hair fibres, the FDSD of sheep with *KRTAP36-1* allele *C* tended to be greater.

## 4. Discussion

This study revealed variation in *KRTAP36-1* in Chinese Tan lambs. When compared with the variation reported previously in New Zealand Merino × Southdown-cross sheep [14], the same three alleles were confirmed to be present, despite the breed differences.

The frequencies of the *KRTAP36-1* alleles were also similar in the two studies. Gong et al. [14] reported frequencies for allele *A* of 14.1%, for allele *B* of 33.7%, and for allele *C* of 52.2%, while in this study, we found frequencies of 12.4% for allele *A*, 29.1% for allele *B*, and 58.5% for allele *C* in the Tan lambs. While breeding history does not record the events that led to the development of the Southdown breed (it is recorded as sheep native to the South Downs of England [18]), the Merino breed (originating from Spain [19], albeit potentially originating from North Africa), and the Chinese Tan breed (endemic to China), the similarities observed in both the nucleotide sequences and allele frequencies of the *A*, *B*, and *C* alleles are rather surprising. This suggests that the genetic variation in *KRTAP36-1* either originated prior to the divergence of these breeds, and/or that evolutionarily the gene and variation therein offer little advantage, and thus historically have not been subjected to either natural or artificial selection (i.e., have not been of benefit to sheep breeding). The conservation of these alleles across genetically distinct breeds may imply functional neutrality or stabilising selection acting on this locus. Alternatively, these alleles might be genetically linked to other loci under selection, indirectly preserving their frequency in different populations.

The HWE test results indicate that the distribution of genotypes in this population is in equilibrium, with no significant deviation between the observed and expected genotype frequencies under HWE assumptions. This suggests that the population of Tan sheep studied is stable and that in the absence of external interferences, including the on-going maintenance of the flock studied, the genotype frequency in this population is likely to remain stable in the future. Further research is needed to confirm this observation. Unfortunately, Gong et al. [14] did not calculate HWE in the New Zealand Southdown × Merino-cross lambs they studied, but the similarity of the allele frequencies in these two studies would suggest that they too were in HWE. It would be useful for future studies to evaluate HWE across a wider range of flocks and environments to determine whether these patterns persist under different selection pressures.

The association detected here suggests that variation in *KRTAP36-1* affects MFC, but this effect is only seen in fine wool fibres and not in heterotypic hair fibres. Given its effect on MFC, it might also affect related fibre traits such as crimp and bulk [20]. How this manifests is unknown, but HGT-*KRTAPs* have been revealed to be differentially expressed in the para- and ortho-cortex of wool fibres, with reports revealing that the KAP6, KAP7 and KAP8 families are preferentially expressed in the ortho-cortex [4]. While the expression of *KRTAP36-1* has not been investigated, if this gene is differentially expressed in these the para- and ortho-cortex cells in a similar manner to other HGT-*KRTAPs*, its expression may lead to differences in the composition, structure or behaviour of the para- and ortho-cortical cells. In this respect, studies have demonstrated that in felting lustre mutant Merino wool, which lacks crimp, the HGT-*KRTAPs* are down-regulated, suggesting that the expression of HGT-*KRTAPs* affects wool crimp [21]. Additionally, in fine wool, the ortho- and para-cortex cells are organised in a distinct bilateral arrangement, which is thought to contribute to crimp characteristics, while for coarse wool (higher MFD wool), this distinct bilateral arrangement is much less obvious [22,23]. This anatomical distinction likely influences the biomechanical bending properties of fibres and provides a potential mechanism by which *KRTAP36-1* variation could result in differences in fibre curvature. In this context, it is perhaps not surprising that the effect is only seen for the fine wool of the Chinese Tan lambs, but not for the heterotypic hair fibres from those same lambs. Although an association with MFC was detected in fine wool fibres, the overall similarity in allele frequencies across breeds suggests that this variation has not been a major target of past selection, possibly due to the modest or fibre-type-specific nature of its effect.

The gene *KRTAP36-1* is clustered with other genes [24,25], and the two *KRTAPs* that are closest to *KRTAP36-1* on ovine chromosome 1 are *KRTAP20-1* and *KRTAP36-2*, located on either side. On one side, *KRTAP20-1* is about 52.2 kb from *KRTAP36-1*, while on the other side, *KRTAP36-2* is close and approximately 7.9 kb from *KRTAP36-1* [24]. Variation in *KRTAP20-1* has previously been linked to variation in wool yield (clean fleece weight/greasy fleece weight × 100%) and MFD in New Zealand Merino × Southdown-cross sheep [25], while variation in *KRTAP36-2* has been associated with variation in wool yield [24]. This suggests the effect of *KRTAP36-1* on wool curvature is unique or specific to just this gene, and not because of its chromosomal linkage to another gene in close proximity to it.

Due to the aggregation of the ovine *KRTAPs* in clusters [26], it cannot, however, be ruled out that there may be other, yet to be identified, *KRTAPs* near *KRTAP36-1*, and that one or more of these may affect wool fibre curvature. This would suggest that further genome or specifically haplotype sequencing is required along with genetic studies too. These should focus on (1) understanding what relationships exist between *KRTAP36-1* and wool traits in other breeds, and (2) providing more haplotype-specific and well annotated genome sequence detail, over extended regions of the families of genes that surround *KRTAP36-1.*

Different *KRTAPs* has been reported to have different effects on the wool traits of Chinese Tan sheep. For example, variation in *KRTAP6-1* has been related to the straightened fibre length at birth and at Er-mao (approximately 35 days postpartum), and with the number of crimps and the degree of crimping at Er-mao [27]. Variation in *KRTAP8-1* is reported to affect the uniformity of Tan lamb wool fibre diameter as measured by the CVFD [28]. Like *KRTAP6-1*, variation in *KRTAP8-2* has also been associated with the degree of crimping of fibres and the length of the straightened fibres at Er-mao [29]. Variation in *KRTAP20-1* has been associated with increased MFC in Chinese Tan lambs [30], and variation in *KRTAP19-5* has also been related to variation in MFC [31]. While *KRTAP6-1* and *KRTAP8-1* have been reported to be differentially expressed in the ortho-cortex [32], the expression patterns of *KRTAP8-2*, *KRTAP20-1* and *KRTAP19-5* have not yet been determined, and further research is needed to better understand the genetic complexity of this class of keratin-associated proteins.

Gong et al. [14] reported that variation in *KRTAP36-1* affected wool PF. Unfortunately, PF was not calculated for the wool fibres of the Chinese Tan lambs studied here, but since the fine wool fibres of the Chinese Tan lambs were of lower MFD than the New Zealand Merino × Southdown-cross sheep studied by Gong et al. [14], one might not expect PF to be as pronounced. Whether an association could be found, if tested, remains to be determined. Future investigations should incorporate a broader range of wool traits, including PF, and involve diverse sheep breeds raised under varying farming systems to determine whether *KRTAP36-1* consistently influences wool characteristics across different genetic backgrounds and environmental conditions.

Overall, this study contributes to the growing understanding of the various roles of *KRTAP* genes in regulating wool fibre traits. It reveals that although these genes are clustered together, they may have distinct effects on different fibre traits, and that these effects can vary across different sheep populations. These findings highlight the need complexity of *KRTAP* function and support the need for further research to characterise the full *KRTAP* repertoire in sheep and to explore their potential influence on a wider range of wool and hair characteristics.

## 5. Conclusions

This study found that there was an association between specific alleles of the gene *KRTAP36-1* and variation in wool trait of Chinese Tan lambs, specifically with variation in the mean fibre curvature of their fine wool fibres. This discovery may contribute to the development of genetic markers, which could then be used to better screen, identify, and select breeding stock with increased or decreased fibre curvature. This should be part of a greater effort to understand the many proteins (both KAPs and keratins) found in wool fibres that appear to affect fibre traits and may ultimately facilitate the breeding of sheep and production of wool that is less variable and better suited for high-quality yarns and fabrics.

## Figures and Tables

**Table 1:** Allele and genotype frequencies for the *KRTAP36-1* gene in the Tan lambs.

Allele/Genotype	Frequency (%)
*A*	12.4
*B*	29.1
*C*	58.5
*AA* (*n* = 5)	2.0
*AB* (*n* = 17)	6.9
*AC* (*n* = 34)	13.8
*BB* (*n* = 22)	8.9
*BC* (*n* = 82)	33.4
*CC* (*n* = 86)	35.0

**Table 2:** Association of *KRTAP36-1* alleles with the four measured fibre traits for the fine wool fibres and heterotypic wool fibres of Chinese Tan Lambs.

Wool Type	Trait ^1^	Allele ^2^	Mean ± SE ^3^		*p*
			Absent	Present	
Fine wool	MFD (μm)	*A*	16.6 ± 0.18	16.8 ± 0.24	0.555
		*B*	16.6 ± 0.19	16.7 ± 0.20	0.518
		*C*	17.0 ± 0.26	16.6 ± 0.17	0.144
	FDSD (μm)	*A*	4.2 ± 0.12	4.1 ± 0.17	0.288
		*B*	4.1 ± 0.13	4.3 ± 0.14	0.102
		*C*	4.2 ± 0.18	4.2 ± 0.12	0.876
	CVFD (%)	*A*	25.2 ± 0.57	24.1 ± 0.78	0.137
		*B*	24.5 ± 0.60	25.6 ± 0.65	0.088
		*C*	24.7 ± 0.85	25.0 ± 0.56	0.691
	MFC (^o^/mm)	*A*	63.9 ± 1.15	63.6 ± 1.58	0.581
		*B*	64.1 ± 1.21	63.5 ± 1.32	0.661
		*C*	61.2 ± 1.70	64.4 ± 1.12	** 0.041 **
Heterotypic wool	MFD (μm)	*A*	29.7 ± 0.34	29.5 ± 0.48	0.742
		*B*	29.7 ± 0.36	29.6 ± 0.40	0.808
		*C*	29.5 ± 0.52	29.7 ± 0.34	0.668
	FDSD (μm)	*A*	8.3 ± 0.15	8.1 ± 0.21	0.431
		*B*	8.2 ± 0.16	8.3 ± 0.17	0.587
		*C*	7.9 ± 0.23	8.3 ± 0.15	0.070
	CVFD (%)	*A*	27.9 ± 0.45	27.6 ± 0.64	0.567
		*B*	27.7 ± 0.48	28.1 ± 0.52	0.434
		*C*	27.0 ± 0.69	28.0 ± 0.44	0.120
	MFC (^o^/mm)	*A*	46.6 ± 0.76	46.6 ± 1.07	0.966
		*B*	46.7 ± 0.80	46.5 ± 0.88	0.786
		*C*	46.3 ± 1.15	46.7 ± 0.75	0.737

^1^ MFD—mean fibre diameter; FDSD—fibre diameter standard deviation; CVFD—coefficient of variation in fibre diameter; MFC—mean fibre curvature. ^2^ Allele *A* was present in 56 lambs and absent in 190 lambs; allele *B* was detected in 121 lambs and absent in 125 lambs; and allele *C* was found in 202 lambs and absent in 44 lambs. ^3^ The predicted means and standard errors of those means derived from GLMs, with *p* value < 0.05 shown in bold.

## Data Availability

The raw data supporting the conclusion of this article will be make available by the authors on request.
